# Preparation and Properties of Low-Exothermic Polyurethanes Doped with Modified Hydrated Salt Phase Change Materials

**DOI:** 10.3390/molecules30071508

**Published:** 2025-03-28

**Authors:** Song Xin, Mengya Sun, Shangxiao Liu, Xuan Zhang, Han Liu

**Affiliations:** 1 College of Transportation, Shandong University of Science and Technology, Qingdao 266590, China; xinsong@sdust.edu.cn (S.X.); hanliu0308@163.com (H.L.); 2 College of Safety and Environmental Engineering, Shandong University of Science and Technology, Qingdao 266590, China; m19861830499@163.com (M.S.); liushangxiao@sdust.edu.cn (S.L.)

**Keywords:** oligothermic, polyurethane, Na_2_HPO_4_-12H_2_O, pyrogenic silica, flame retardant

## Abstract

In this study, fumed silica (FS) was used as a support material and infused with the hydrated salt sodium hydrogen phosphate dodecahydrate (DHPD) to create shape-stabilized constant phase change materials (CPCMs). These CPCMs were integrated into a polyurethane matrix as a functional filler, resulting in low-exothermic polyurethane composite foams (CPCM-RPUFs) that demonstrate thermoregulation and flame-retardant properties. Recent findings show that CPCM-RPUF excels in thermal stability compared to pure polyurethane, with a melt phase transition enthalpy of 115.8 J/g. The use of fumed silica allows for the encapsulation of hydrated salts up to 87%, ensuring the structural integrity of the vesicles. As FS content in CPCMs increased, the internal temperature of the composite foam significantly decreased, showing excellent thermal regulation. Thermogravimetric analysis showed that the synergistic effect of DHPD and FS improved the thermal stability and flame retardancy of the composites. By monitoring the internal and surface temperature changes in the foam, it was verified that CPCMs can effectively alleviate heat accumulation during the curing process and reduce the core temperature (56.9 °C) and surface warming rate, thus realizing the thermal buffering effect. With the increase in FS content in CPCMs, the compressive strength of CPCM-RPUF can be maintained or even enhanced. This study provides a theoretical basis and technical support for the development of polyurethane composite foams with integrated thermal regulation and flame-retardant properties, which can have broad application prospects in the fields of building energy conservation, energy storage equipment, and thermal mine insulation.

## 1. Introduction

Rigid polyurethane foam (RPUF) offers several advantages, including low thermal conductivity, low density, good dimensional stability, corrosion resistance, and effective bonding with various materials. As a result, it is widely used in building insulation [1], tunnel insulation, refrigeration devices, mine leakage wind plugging, and grouting applications. One of the most effective energy-saving technologies for thermal storage systems is latent heat thermal energy storage (LHTES), which employs phase change materials to store and release thermal energy [2]. This method is currently considered one of the most effective and promising ways to store energy. Rigid polyurethane foam is recognized as an ideal next-generation building insulation material, and there is growing interest in combining polyurethane foam with phase change materials. Research has demonstrated that incorporating an appropriate amount of phase change materials into the polyurethane matrix [3] can enhance thermal energy storage capacity. However, the process of generating polyurethane foam generates heat, which creates a fire hazard and limits its potential applications [4]. To mitigate these risks, it is essential to develop a new type of low-temperature polymerized polyurethane that focuses on reducing synthesis temperature while enhancing its flame-retardant properties without compromising its mechanical properties.

In the field of low-temperature polymerization of polyurethanes, a study by Zhang et al. [5] found that the threshold curing reaction temperature of polyurethane foam (PUF) is negatively correlated with the proportion of physical blowing agent (HCFC-141b) or high boiling solvent, and positively correlated with the proportion of water. Additionally, it was observed that the curing temperature threshold of PUF increases with higher density and decreases with lower ambient temperatures. Amundarain et al. [6] demonstrated that the central maximum temperature of PUF progressively decreases as the content of recycled polyols (RPs) increases. Liu et al. [7] developed novel polydopamine (PDA)/SiO_2_/urea-formaldehyde resin (UF) hetero-shell phase change microcapsules (S-MPCMs) and embedded them into composite polyurethane foam (MP/PU) using a one-step method. Their results showed that the internal peak temperature of MP/PU foam decreased to 53.7 °C, demonstrating the excellent thermal regulation properties of S-MPCMs. Qin [8] prepared a novel polyurethane/nano fly ash grouting material through in situ polymerization. The study indicated that the composite achieved an optimal compressive strength of 44 MPa, and the curing temperature was reduced to a maximum of 82 °C when the dosage of nano fly ash was 40% and that of the inorganic hydration salt was 10%.

Incorporation of flame retardants into rigid polyurethane foam (RPUF) by physical or chemical means is a common strategy to enhance flame retardancy. Incorporation of additives such as halogen-, phosphorus-, and nitrogen-based additives can significantly enhance the flame retardancy of the material. Chung [9] prepared rigid polyurethane foam (RPUF) using phosphorus-based flame retardants including TCPP (tri(2-chloropropyl)phosphate), TEP (triethyl phosphate), and TMP (trimethyl phosphate). It was shown that these flame retardants affected the foaming process, cell structure, and mechanical strength of RPUF, but they also increased its suitability as a thermal insulation material. The material exhibited self-extinguishing properties due to its low thermal conductivity, and the oxygen index of all samples exceeded 21 vol%. Wang [10] prepared halogen-free flame-retardant rigid polyurethane foam (RPUF) by combining SiO_2_ nanorods/graphene oxide hybrids with dimethyl methylphosphonate foam. The results showed that the combination of dimethyl methylphosphonate and SiO_2_ nanospheres/graphene oxide greatly enhanced the flame retardancy and mechanical properties of rigid polyurethane foam compared to pure rigid polyurethane foam (RPUF). Lu et al. [2] prepared organic–inorganic hybrid PU using highly dispersed melamine/red phosphorus composite as a filler, and their experimental results showed that the peak heat release rate and the total exothermic rate were reduced by 30.1% and 51.7%, respectively, compared to the pure PU foam. Rao et al. [11] prepared polyurethane foams with flame-retardant and smoke-suppressing properties by combining ethylene glycol and phenylphosphonic dichloride (PPDC), which were blended with expanded graphite and polyether polyol (PPG-2000). In addition, Yu et al. [12] proposed an interfacial bonding and in situ filling strategy to introduce self-expanding biomass aerogel particles into RPUF. It was shown that the thermal conductivity decreased by 21.6% after the introduction of the aerogel particles. The aerogel/RPUF composites exhibited excellent flame retardancy, with an ultimate oxygen index of 28%. Additionally, the peak heat release rate and smoke generation were reduced by 38.0% and 54.0%, respectively, while the compressive strength increased by 93.7%. At this stage, the formulation of polyurethanes is based on the consideration of synthesis temperature or the enhancement of flame retardancy, whereas the actual use of polyurethanes requires the comprehensive consideration of synthesis temperature and flame retardancy, and there are fewer comprehensive studies in this regard.

Phase change materials (PCMs) are crucial in latent heat storage and can be categorized into two main groups: organic and inorganic [13,14]. Hydrated salt, as a high-performance inorganic PCM, has the advantages of a high enthalpy of phase change and low cost [15], but suffers from some inherent drawbacks, such as leakage during phase change [16], subcooling [17], and phase separation [18]. These drawbacks hinder the efficient storage of thermal energy and its susceptibility to subcooling and phase separation in thermal energy storage (TES) limits its further application. Therefore, hydrated salts need to be simultaneously subcooled and encapsulated for more efficient long-term energy storage. In recent years, porous media [19] have been widely used for the modification of hydrated salt composite phase change materials (PCMs) prepared as shape-stabilized PCMs (CPCMs) to enhance their thermal stability. Liu et al. [20] utilized expanded graphite (EG) and expanded graphite oxide (EGO), loaded with sodium carbonate decahydrate (Na_2_CO_3_-10H_2_O) and disodium hydrogen phosphate dodecahydrate (Na_2_HPO_4_-12H_2_O) eutectic hydrated salt (EHS), as support materials; thus, two shape-stabilized PCMs were prepared. The results showed that the confinement effect decreased the phase transition temperatures of both CPCMs; however, due to the abundance of oxygen-containing functional groups on the surface of EGO and the large layer spacing, the eutectic salt had a higher loading capacity and stronger intermolecular forces on its surface, so the EHS/EGO composites had a higher phase transition temperature and a stronger energy storage capacity.

Fumed silica (FS) is a nanomaterial with a porous structure, which has been widely used as a non-metallic porous medium and thickener due to its stable physicochemical properties, small particle size, and large specific surface area. It has superior properties such as high-temperature resistance, chemical inertness, and thickening properties, which make it an ideal material for the modification of PCMs including hydrated salts [21]. Na_2_HPO_4_-12H_2_O (DHPD) is expected to be an ideal hydrated salt for latent heat storage due to its high latent heat and little phase separation. Canbazolu’s study [22] showed that DHPD has a high latent heat at a melting point of 278.84 J/g. However, DHPD also suffers from two major drawbacks: supercooling and phase separation. Tamaru et al. [23] prepared hard-shelled microcapsules containing DHPD and reduced its supercooling, but the cost was too high compared to other methods of modifying DHPD. Xiao et al. [24] introduced gel and sodium alginate into DHPD, and the latent heat of PCM reached 190 J/g, but one of the composites experienced 10% volumetric leakage after 100 freeze–thaw cycles, which is not satisfactory in practical applications. Feng et al. [25] prepared porous FS-based adiabatic composite PCMs and noted that the thermal shrinkage inhibition rate of FS was only 0.01512, which showed good thermal stability and has the potential to support shape-stabilized PCMs with high-quality additives. Yu et al. [26] developed a biobased PCM with FS additives that can be used in building energy-efficient concrete. The weight loss of the FS-added biobased PCM was reduced by more than 27% at temperatures above 300 °C with good thermal stability. Peng [27] developed a novel composite phase change material (PCM) formed by immersing disodium hydrogen phosphate dodecahydrate (DHPD) in fumed silica (FS). The results showed that the uniform distribution of FS effectively prevented the dehydration of DHPD and reduced its supercooling from 14.4 °C to 4.1 °C. After 50 heating-cooling cycles, the phase transition temperature of the composite remained stable with a latent heat of phase transition of 211.8 J/g, which showed excellent thermal stability and proved that the DHPD/FS was an excellent material for thermal energy storage.

Based on the water-absorbent and non-corrosive nature of fumed silica (FS), which can prevent the dehydration of Na_2_HPO_4_-12H_2_O (DHPD) in air and act as a crystal nucleus to reduce the supercooling of hydrated salt, fumed silica (FS) was chosen as a support material in this study for the preparation of shape-stabilized PCMs loaded with the Na_2_HPO_4_-12H_2_O (DHPD) hydrated salt (CPCMs). Low exothermic polyurethane systems with thermal regulation were prepared by mixing CPCMs with compound polyethylene glycol (RPEG) and polymethylene polyphenylene polyisocyanate (PAPI) in different mass ratios under vigorous stirring. Subsequently, the morphology, chemical composition, thermal stability, thermal regulation, and flame-retardant properties of the composite polyurethane materials were systematically characterized, and their potential mechanisms were discussed.

## 2. Experiment and Method

### 2.1. Materials

Polyethylene glycol (PEG) (Mn = 1000; Mn = 1500), polymethylene polyphenylene isocyanate (PAPI), cyclopentane, dimethicone, dimethyl silicon oil, diethylene glycol monohydrate, disodium hydrogen phosphate dodecahydrate (DHPD), and tris(1-chloro-2-propyl) phosphate (TCPP) were purchased fromQingdao Jingke Instrument Reagent Co., Ltd., China, Qingdao, China, and all reagents and solvents were of analytical grade and used as is without further purification.

### 2.2. Preparation of CPCMs

**Figure 1:**
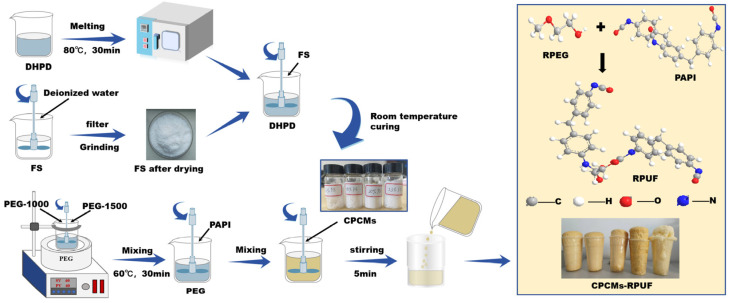
Flow chart of CPCMs and CPCM-RPUF preparation.

CPCMs were prepared with the melt impregnation method. As shown in Figure 1, Na_2_HPO_4_-12H_2_O (DHPD) was impregnated into fumed silica (FS) to form shaped composite phase change materials (CPCMs). A certain amount of FS and deionized water was added to a beaker. The mixture was stirred and sonicated for 30 min, and then the sample was filtered, dried, and ground to obtain FS powder. The preformed FS powders (15%, 18%, 20%, 23%, and 25%) were proportionally mixed with melted DHPD in a beaker and stirred at a temperature of 40 °C. Finally, after homogeneous dispersion and solidification at room temperature, the stereotyped composite phase change materials (CPCMs) were obtained.

**Figure 2:**
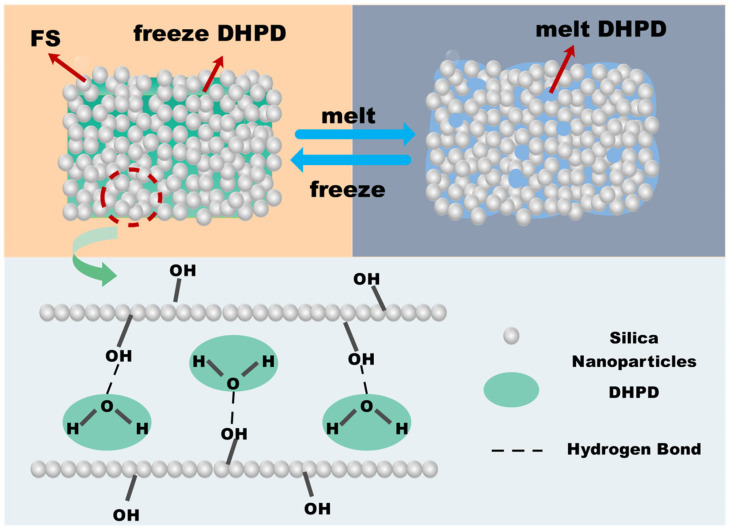
Modulation mechanism of DHPD by fumed silica (FS).

The mechanism of fumed silica (FS) modulation of DHPD is revealed in Figure 2. The fundamental reason why fumed silica-loaded hydrated salt Na_2_HPO_4_-12H_2_O (DHPD) can effectively avoid leakage is due to the existence of a series of weak intermolecular interactions (e.g., hydrogen bonding, capillary force, surface tension, etc.) between porous nano-silica and the hydrated salt [28], as well as the “confinement” effect brought about by the size effect of the pores. On the one hand, the pores form a physical space closure for DHPD, reducing its mobility; on the other hand, the interaction forces such as hydrogen bonding and adsorption between hydrophilic groups on the surface of the pore wall and the hydrated salt molecules can stabilize the DHPD and impede it from escaping from the pores, thus effectively avoiding leakage, which is conducive to the enhancement of the thermal stability and cycling reliability of phase change energy storage materials.

### 2.3. Formatting of Mathematical Components

CPCM-RPUFs were prepared using a one-step method. As shown in Figure 1, polyethylene glycol PEG-1000 and polyethylene glycol PEG-1500 were weighed according to an 8:2 mass ratio and placed sequentially behind a beaker, which was then placed in an oven and melted at 80 °C. The melted sample was removed from the oven and stirred continuously with a stirrer to make the mixture homogeneous, which was cooled and cured, and then dried and dehydrated at 110 °C. The 110 °C dried and dehydrated RPEG, dimethyl silicone oil, monocrystalline diethylene glycol, cyclopentane, TCPP, and CPCMs (5 wt% of 15% FS, 18% FS, 20% FS, 23% FS, and 25% FS) were added to a beaker, stirred for 30 s, and mixed homogeneously and recorded as component A. The sample was then dried and dehydrated at 110 °C. Quantitative PAPI was weighed and recorded as component B. Component A was mixed well, added to component B, and stirred rapidly until bubbles appeared and foaming occurred. In this paper, five different polyurethane phase change materials, named CPCM-RPUF-1, CPCM-RPUF-2, CPCM-RPUF-3, CPCM-RPUF-4, and CPCM-RPUF-5, were prepared based on five different ratios of CPCMs following the same experimental steps.

### 2.4. Testing and Characterization

All prepared CPCMs were tested for leakage by heating the samples to 60 °C for 15 min and then to 80 °C for 30 min. The surface morphology and microstructure of the CPCMs and CPCM-RPUFs were analyzed using field emission SEM (APREO, Thermo Fisher Scientific, Waltham, MA, USA). The phase transition temperatures and latent heats of the CPCMs and CPCM-RPUFs were obtained using a MettlerDSC1 calorimeter (Mettler Toledo, Zurich, Switzerland) under nitrogen at a heating and cooling rate of 5 °C/min and a nitrogen scan rate of 50 mL/min. The thermal stability of CPCMs and CPCM-RPUFs was investigated using a thermogravimetric analyzer (Mettler Toledo, Zurich, Switzerland) under a nitrogen atmosphere at a heating rate of 10 K^−1^min^−1^. The chemical composition of CPCM-RPUFs was analyzed using FTIR (Nicolet iS50, Thermo Fisher Scientific, Waltham, MA, USA). Type K thermocouples were arranged at the center of the mold to collect the temperature values in the core area of the foam, and the data were recorded by a rover, with the collected data imported into a computer for analysis. Cylindrical samples of dimensions Φ50 × 100 were prepared using a homemade mold, and uniaxial compression tests were performed using a Shimadzu electronic universal material testing machine (AG-X250, Kr, Shanghai, China).

## 3. Discussion

### 3.1. Shape Stability

**Figure 3:**
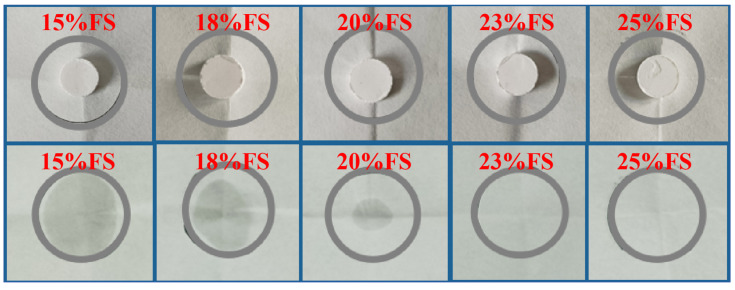
Leakage test of CPCMs at different mass fractions of FS.

The loading of the porous material on the phase change material always has its maximum value, and the leakage of the phase change material occurs when the loading exceeds the adsorption amount of the porous material, so the maximum adsorption amount of FS should be determined according to the leakage experiment. Figure 3 below shows the photos of composite phase change materials with different contents before and after heating; as shown in the figure, the shaped composite phase change materials before heating present different states because of the different contents of FS, due to the strong hydrophilicity of silica, and the surface of the composite material column with only 15% FS added is relatively wet, while, with the increase in FS content, the surface of the material column becomes dry and uneven. After heating, the composites with 15%, 18%, and 20% FS show leakage, the traces of which gradually decrease with the increase in FS content, and no phase change material is found to leak out of the filter paper at ratios of 23% and 25%, which successfully prevent the leakage of phase change material, with the minimum content of FS to prevent the leakage of material obtained to be 23, i.e., the maximum adsorption capacity of FS for DHPD PCMs is 87%. The maximum adsorption amount is 87%.

### 3.2. Thermal Storage Properties of CPCMs

**Figure 4:**
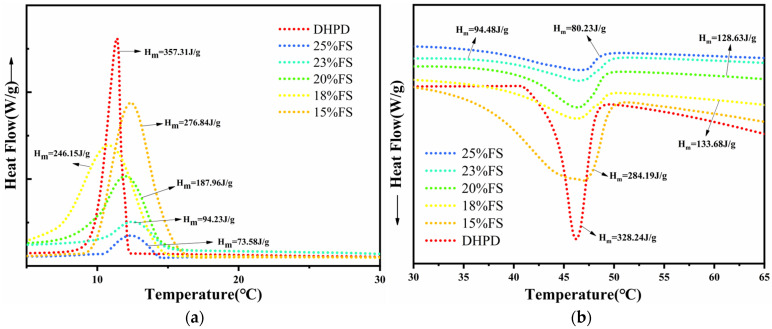
Cooling curves (**a**) and melting curves (**b**) of DHPD and CPCMs containing different mass fractions of FS.

The addition of FS causes changes in the phase transition temperature and enthalpy of DHPD, and the amount added also affects the magnitude of the changes. For the enthalpies of phase transition of the complexes, the addition of FS decreases all the enthalpies of phase transition of the complexes, which is related to the inability of FS to generate the latent heat of phase transition. From Figure 4, it can be seen that the enthalpies of phase transition of the prepared CPCMs are 152.19 J/g, 79.68 J/g, 75.43 J/g, 76.23 J/g, and 54.63 J/g when the mass fractions of FS are 15, 18, 20, 23, and 25%, respectively. It can be seen from the DSC melt curves that the phase transition peaks of the hydrated salts are broadened as well, and the peak positions are shifted forward, which may be related to some extent to the limitation of phase change materials within the pores [29,30]. In the FS with a larger pore size, the difference between the phase transition temperature and that of the hydrated salt material in the conventional state is very small, and the effect on the temperature regulation of the hydrated salt is obvious when the carrier microporous content and specific surface area are higher. When a small amount of FS is added to the hydrated salt, the hydrated salt is in a saturated state, and the FS has a limited effect on the crystalline water in the hydrated salt, thus its cooling effect is not obvious; on the contrary, when the FS is in sufficient quantity, the hydrogen bonding effect on the crystalline water and the capillary effect are enhanced, weakening the force of DHPD on the water molecules, and thus the thermoregulation effect is obvious.

### 3.3. Microscopic Morphology of CPCMs

**Figure 5:**
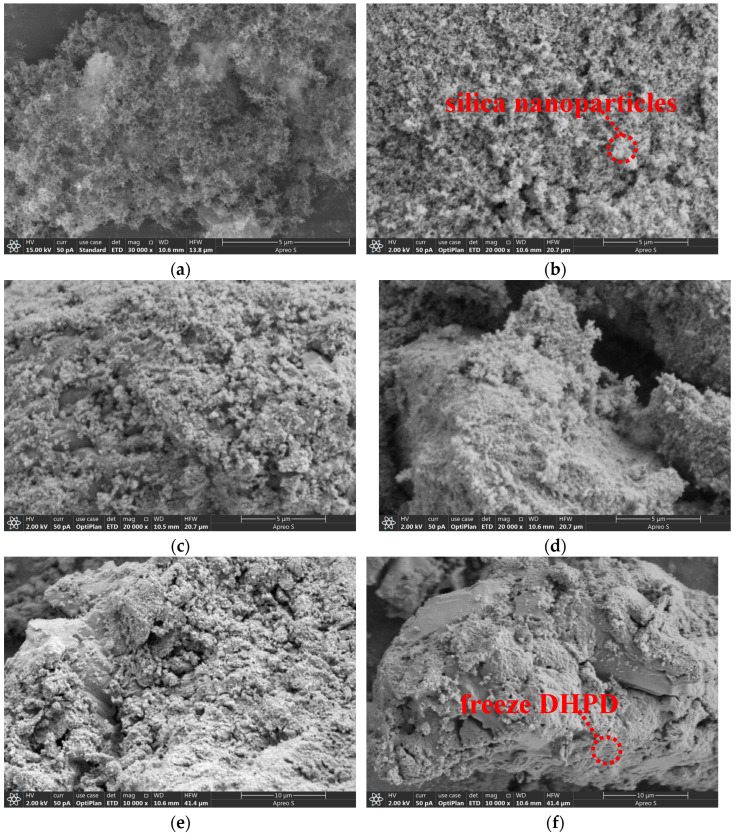
SEM images of CPCMs containing different levels of FS: (**a**) pure FS, (**b**) 15% FS, (**c**) 18% FS, (**d**) 20% FS, (**e**) 23% FS, and (**f**) 25% FS.

Figure 5 shows the microstructures on the surfaces of FS and CPCMs. From Figure 5a–f, it can be seen that FS is a loose three-dimensional porous structure stacked by a large number of aggregated spherical SiO_2_ particles, and the formed pores have the sizes of macropores and mesopores. There are also a few larger SiO_2_ particles with irregular shapes in the figure, and none of the particles have obvious cracks and broken pores. As the fumed silica itself is full of micropores and mesopores, these pores can enhance the adsorption capacity of FS, and the capillary effect is used to allow DHPD to fill stably into the inter-pore spaces. After the integration of DHPD and FS, as can be seen in Figure 5, the spherical SiO_2_ particles can still be observed, stacked on the microscopic morphology of the CPCMs, but the pores formed by the stack have been filled with the hydrated salt crystalline material, forming a dense bulk structure. This indicates that the DHPD has been effectively and uniformly adsorbed and filled into the FS pores under the effect of hydrogen bonding, capillary force, and surface tension. In this composite, the FS can effectively support and shape DHPD to prevent leakage.

### 3.4. Microscopic Morphology of CPCM-RPUFs

**Figure 6:**
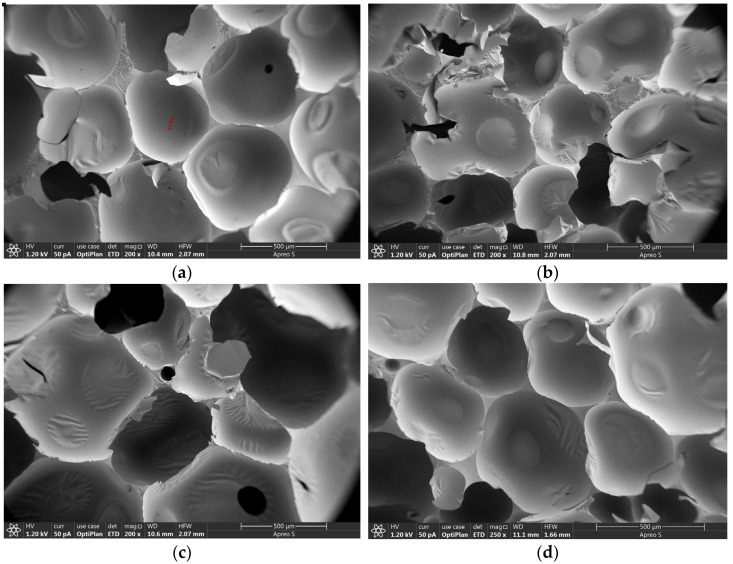

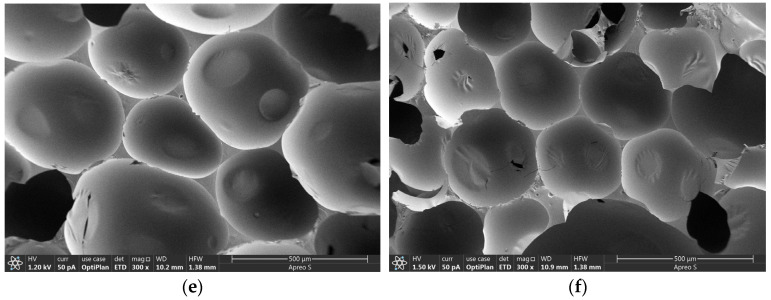
SEM images of composite foams containing different contents of CPCMs: (**a**) pure PU foam, (**b**) CPCM-RPUF-1, (**c**) CPCM-RPUF-2, (**d**) CPCM-RPUF-3, (**e**) CPCM-RPUF-4, and (**f**) CPCM-RPUF-5.

The morphology and microstructure of the pure PU foams and CPCM-RPUFs were characterized by SEM. As can be seen in Figure 6, the pure PU foams exhibited a typical closed-cell structure, with the shape of the vesicles close to a spherical shape, the size of the vesicles ranging from 256.32 μm to 340.71 μm, and the average vesicle size of 304.83 μm, and the vesicles were uniformly sized with regular distribution and orderly arrangement. The incorporation of CPCMs as phase change materials into the polyurethane matrix may hurt the shape and size of the PU. As shown in Figure 6, CPCMs were well dispersed in the polyurethane matrix, and no particle agglomeration was observed. The CPCM-RPUF still had a closed-cell structure with near-spherical vesicles, but the vesicle homogeneity was reduced. As shown in Figure 6b, the diameter of the vesicles of the composite foam increased significantly and a large number of collapsed cells were generated compared to that of the pure PU foam. This is due to the polyurethane curing process generating a large amount of reaction heat, resulting in the leakage of CPCMs, DHPD’s constant heat shedding of crystalline water, and the isocyanate group of the PAPI reaction; the increase in crystalline water leads to foaming, and the gel reaction cannot reach equilibrium, so the rate of CO_2_ generation is fast, the small molecules of CO_2_ gas sealed in the matrix escape, and the volume expands, which results in the an increased tendency of bubble holes, macroscopically manifested as a decrease in the strength of the solidified body. As shown in Figure 6d,e, the distribution of vesicles in composite foams is more uniform and fine, which may be due to the better encapsulation and stabilization effect of higher FS content materials, which can reduce the risk of an excessive release of water from DHPD to a certain extent and thus avoid the collapse and expansion of vesicles caused by the rapid escape of a large amount of CO₂. CPCMs can inhibit the detachment of a large amount of water from crystalline water in the process of phase transition and the excessive reaction with PAPI, which can lead to a decrease in the strength of the solid body on a macro scale. CPCMs can inhibit the detachment of crystalline water and the excessive reaction with PAPI during the phase change process, thus effectively avoiding the deformation or rupture of the vesicles and ensuring the integrity and stability of the vesicle structure.

### 3.5. Chemical Structure of CPCM-RPUFs

**Figure 7:**
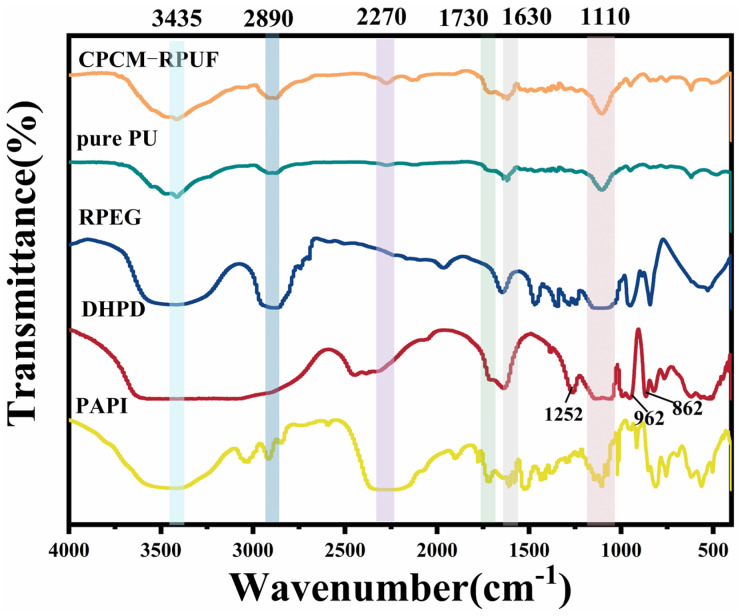
FT−IR spectra of pure PU, CPCM-RPUF, RPEG, DHPD, and PAPI.

**Figure 8:**
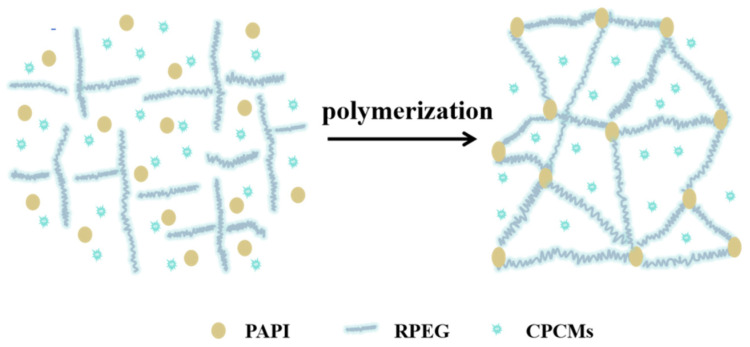
Schematic route of the CPCM-RPUF reaction.

To evaluate the chemical composition of the samples, the FT-IR spectra of pure PU, CPCM-RPUF, RPEG, and DHPD were compared, as shown in Figure 7. In the IR spectra of PU, CPCM-RPUF, RPEG, DHPD, and PAPI, relatively broad peaks appeared near the range of 3435 cm^−1^, which belonged to the peaks of the telescopic vibration of the -OH group in the materials [7]. The peaks at around 1635 cm^−1^ corresponded to the stretching vibration of the OH groups of water [7,31,32,33]. In the absorption spectrum of RPEG, the peak at 1110 cm^−1^ corresponds to the C-O stretching vibration, while in both pure PU and CPCM-RPUFs, the C-O absorption peak of RPEG appeared at 1110 cm^−1^, which indicated that RPEG and PAPI had successfully synthesized polyurethanes by chemical reaction. In the IR spectral region of DHPD, the absorption peaks at about 1252, 1110, 962, and 862 cm^−1^ were due to the antisymmetric telescopic vibration peak of P-O and the symmetric telescopic vibration peak and telescopic vibration peak of P-OH, respectively. A distinct PAPI band was observed at 2270 cm^−1^, which corresponds to the asymmetric stretching vibration of -NCO. In CPCM-RPUF, the characteristic absorption peak of -NCO was observed near 2270 cm^−1^, which indicated that some isocyanate failed to react with a polyol in time after the rapid reaction of CPCM-RPUF, but the disappearance of the characteristic absorption peak of -NCO in the pure PU indicated that PAPI in the raw material was completely reacted, and it could be deduced that the crystallization water attached to the DHPD leaked and reacted with -NCO, consuming part of the isocyanate. Meanwhile, the characteristic absorption peak of the carbonyl group (C=O) was observed at 1730 cm^−1^, along with additional peaks of C-N and N-H, indicating that the hydroxyl group (-OH) and -NCO (in PAPI) reacted successfully to form a carbamate group (-NH-COO-). The schematic route of the CPCM-RPUF reaction is shown in Figure 8, where the CPCMs and the urethanes were only mixed physically, and no chemical reaction was involved. The FTIR spectra of CPCM-RPUF showed the same characteristic absorption peaks as that of pure PU and no new absorption peaks appeared by comparing pure PU with CPCM-RPUF, consistent with the results in Figure 7.

### 3.6. CPCM-RPUF Thermal Stability and Thermal Storage Performance

**Figure 9:**
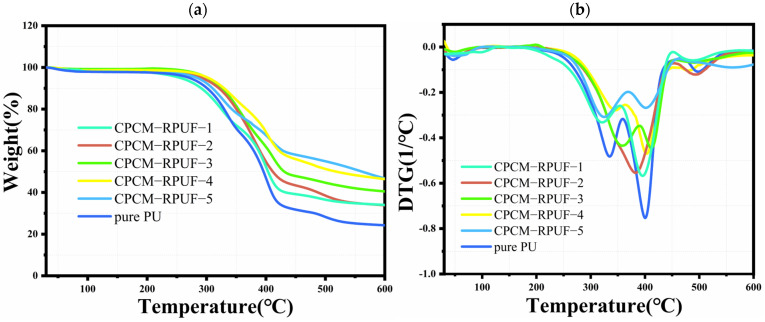
TG curve (**a**) and DTG curve (**b**) of CPCM−RPUF.

Thermal stability is an important indicator for evaluating the service life of materials, which is directly related to their practical application value [34]. Therefore, it is important to determine the resistance of these materials to degradation at high temperatures. For this purpose, thermogravimetric analysis was carried out and two curves of mass loss with increasing temperature (TG curves) and their first-order derivatives’ DTG curves were obtained, which are shown in Figure 9a,b. In addition, the temperatures T_5%_ °C, T_10%_ °C, and T_50%_ °C are given in Table 1, which correspond to the temperatures at 5%, 10%, and 50% weight loss of the analyzed samples and also include the three maxima of the rate of mass loss (T_DTGmax1_, T_DTGmax2_, T_DTGmax3_). Based on the obtained TG curves, it can be seen that the weight loss of both pure PU and CPCM-RPUF was insignificant at temperatures up to 200 °C, indicating their good thermal stability at common operating temperatures. The mass loss in this temperature range is related to the evaporation of water and other unreacted monomers remaining in the sample [35,36]. Starting from a temperature of about 350 °C, a higher thermal resistance is observed for the reference samples containing CPCMs. Analyzing the TG and DTG plots, it can be seen that the weight loss process of polyurethane is divided into three stages. The first stage is in the range of ~300–350 °C, and the weight loss in this stage is mainly attributed to the initial pyrolysis of the hard chain segments of PU; the second stage is in the range of 350–450 °C, when the soft chain segments of the PU start to decompose and are accompanied by further pyrolysis [37]; and the third stage, which is above 450 °C, is characterized by the gradual decomposition of the residual matrix, which exhibits a slow weight loss. Compared with pure PU, the initial temperature of thermal decomposition of CPCM-RPUF-1 was slightly lower, and the initial degradation temperature was about 10–15 °C lower than that of pure PU. This may be attributed to the leakage of hydrated salts leading to a slight decrease in the thermal stability of the soft-chain segment of the PU matrix. In addition, since FS has a high melting temperature and is difficult to degrade at higher temperatures, the residual amount of CPCM-RPUF at 600 °C increased gradually with the increase in the addition of CPCMs, which suggests that the incorporation of CPCMs has a positive effect on the enhancement of the overall thermal stability of PU. Zhang et al. [38] investigated the thermal stability of foams with nano-SiO_2_. The study obtained a higher amount of residue (20%), which indicated that the inorganic fillers improved the thermal stability of the material. Therefore, the addition of CPCMs can improve the thermal stability of polyurethane systems to some extent.

**Figure 10:**
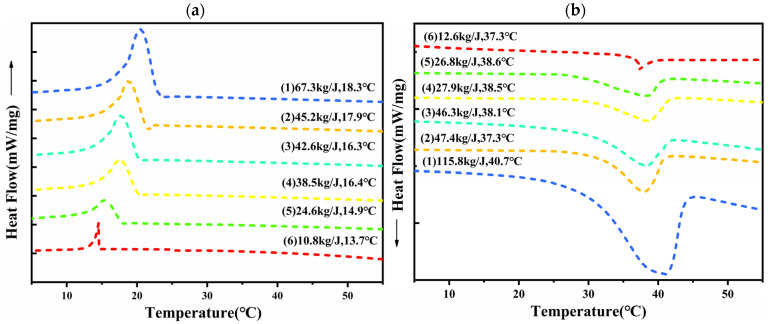
Cooling curves (**a**) and melt curves (**b**) of pure PU and CPCM-RPUF: (1) CPCM-RPUF-1, (2) CPCM-RPUF-2, (3) CPCM-RPUF-3, (4) CPCM-RPUF-4, (5) CPCM-RPUF-5, and (6) pure PU foam.

The phase transition properties of phase change materials are crucial for the effective application of phase change materials in energy storage [28]. Figure 10 shows the DSC curves of pure PU and CPCM-RPUF. As shown in Figure 10, the phase transition temperature of the composites gradually increased from 37.3 °C to 40.7 °C and the enthalpy of phase transition significantly increased from 12.6 J/g to 95.8 J/g with the increase in CPCMs doping ratio, indicating that the doping of CPCMs significantly enhances the energy storage capacity and thermal regulation performance of the materials. This is mainly attributed to the phase transition properties of CPCMs in the matrix material and the effective encapsulation of the hydrated salt by fumed silica (FS), which ensures the thermal stability and phase transition capability of the CPCM-RPUF composites during thermal cycling.

### 3.7. Research on Temperature Regulation Performance and Flame-Retardant Mechanism of CPCM-RPUF

**Figure 11:**
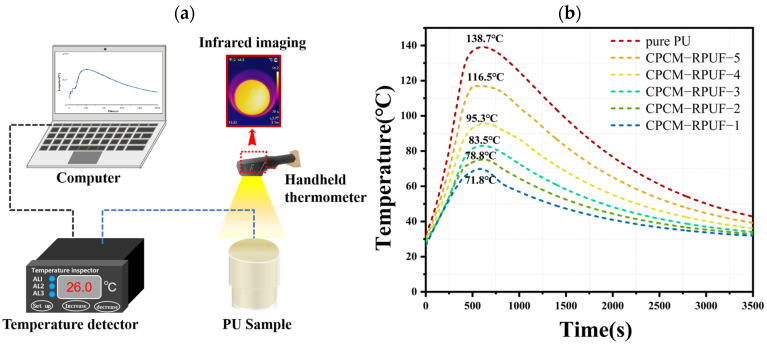
Experimental set-up diagram (**a**) and temperature/time profiles for pure PU and CPCM-RPUF (**b**).

**Figure 12:**
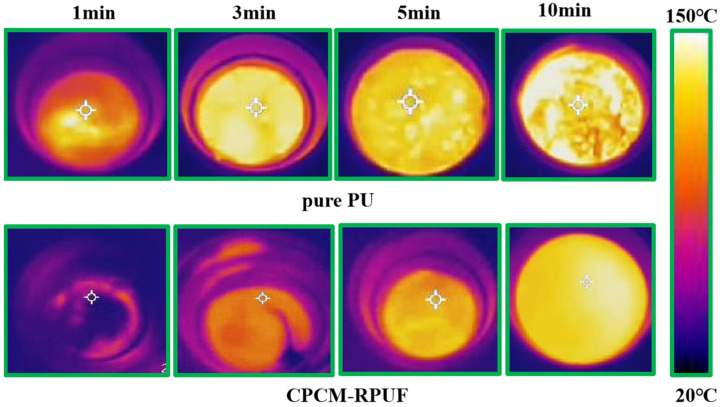
Surface infrared thermography images of pure PU and CPCM-RPUF.

The temperature change at the center point inside the CPCM-RPUF was tested on the experimental platform as shown in Figure 11a (the temperature change after the addition of CPCMs to PU was recorded). Analyzing Figure 11b shows that the temperature of pure PU is higher than that of CPCM-RPUF. This can be attributed to the continuous accumulation of heat during the curing process of the pure PU, which led to a rapid increase in the temperature of the core area, with a maximum temperature of 138.7 °C recorded. The internal temperatures of the CPCM-RPUF were 71.8, 78.8, 83.5, 95.3, and 116.5 °C when blended with CPCM contents of 25% FS, 23% FS, 20% FS, 18% FS, and 15% FS, respectively. The significant decrease in temperature is because the hydrated salt phase change material (DHPD) absorbs a portion of the heat released by the foam during the melting process. This leads to a reduction in the degree of temperature increase within the core area. Using a thermal imager, it was possible to monitor the temperature changes in the CPCM-RPUF material, with the temperature differences recorded on the surface of the specimen indicated by different colors. As can be seen in Figure 12, in the case of pure PU, heat is continuously released during the foam formation process. The heat is continuously conducted to the surface of the specimen, resulting in a rapid increase in temperature. The rate of increase in surface temperature of the CPCM-RPUF is significantly slower than that of the pure PU. There is a significant temperature hysteresis phenomenon in the CPCM-RPUF system, and the degree of significance of the temperature hysteresis phenomenon increases with the decrease in the FS content of CPCMs. The CPCM-RPUF has good thermal regulation properties in PU foam, which can be attributed to the good distribution characteristics of CPCMs in the foam.

**Figure 13:**
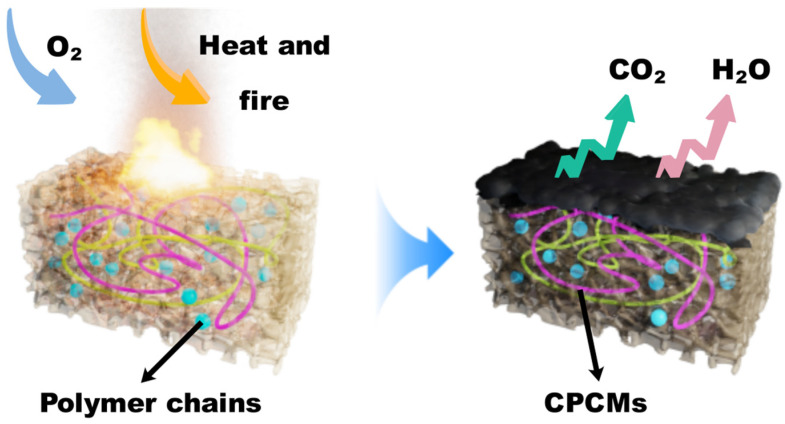
CPCM-RPUF flame-retardant mechanism diagram.

The polyurethane foaming process tends to generate a lot of heat and has a certain tendency to fire. By incorporating hydrated salt-based CPCMs into the PU, a phase transition can occur to absorb part of the heat and reduce the accumulation of heat in the core area of the PU. As shown in Figure 13, even under the influence of an external ignition source, the thermal decomposition of CPCMs and RPEG releases a large amount of CO_2_ and water vapor, etc., which dilutes the concentration of O_2_ and absorbs a large amount of heat, suppressing further combustion of the material. In addition, the silicon hydroxyl structure on the surface of FS is rapidly transformed into a Si-O-Si network structure when heated, which forms a protective film on the surface of the specimen [7]. This protective film effectively inhibits the thermo-oxidative degradation reaction of the coke layer and acts as a supporting skeleton on the surface of the carbon layer, which significantly enhances the structural stability of the carbon layer and effectively blocks the reaction process inside the material.

### 3.8. Compression Properties of CPCM-RPUFs

**Figure 14:**
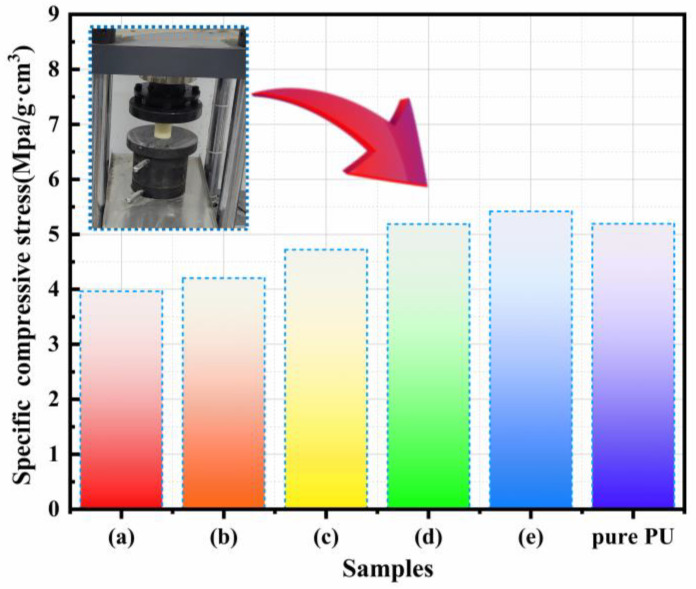
Effect of CPCMs on the compressive strength of CPCM-RPUF: (a) CPCM-RPUF-1, (b) CPCM-RPUF-2, (c) CPCM-RPUF-3, (d) CPCM-RPUF-4, (e) CPCM-RPUF-5.

The effect of CPCMs on mechanical properties was investigated with a uniaxial compression test and the results are shown in Figure 14. It can be observed that the specific compressive strength of (a) CPCM-RPUF-1, (b) CPCM-RPUF-2, and (c) CPCM-RPUF-3 decreased compared with that of pure PU, which is due to the leakage of phase change materials in CPCMs, resulting in larger pore size of the foams, the rupture of the vesicles, and the macroscopic manifestation of the decrease in the strength of the solids, which is in agreement with the characterization image of SEM. However, the specific compressive strength of (d) CPCM-RPUF-4 and (e) CPCM-RPUF-5 is equal to or even exceeds that of pure PU, which is because the addition of 23% and 25% of FS in CPCMs can successfully prevent the leakage of phase change materials, thus effectively avoiding the deformation or rupture of the foam pores and guaranteeing the integrity and stability of the foam pore structure, which is in line with the results of leakage experiments.

## 4. Conclusions

In this paper, shape-stable phase change materials (CPCMs) were prepared using fumed silica (FS) as a support material and loading Na_2_HPO_4_-12H_2_O (DHPD) hydrated salt. Subsequently, low-exothermic polyurethane systems with thermal regulation and flame-retardant properties were prepared with a one-step method using CPCMs as fillers, and the comprehensive performance and flame-retardant mechanism of CPCMs and CPCM-RPUF were systematically analyzed.

1. The enthalpy of the melt phase transition of the prepared CPCMs reached the highest value of 152.19 J/g. The FS encapsulated DHPD by 87%, which could effectively support and set DHPD and prevent leakage.
2. Compared with pure PU, the phase transition temperature and maximum weight loss temperature of CPCM-RPUF moved to higher values, showing better thermal cycling ability and thermal stability. Even under the influence of external ignition sources, the synergistic effect of DHPD and Si-O-Si could enhance the stability of the carbon layer of the foam and improve the flame-retardant property of CPCM-RPUF.
3. The CPCM-RPUF system has an obvious temperature hysteresis phenomenon, and the significant degree of the temperature hysteresis phenomenon increases with the decrease in the FS content in CPCMs, which demonstrates the good distribution characteristics and thermal buffering effect of CPCMs in the PU matrix.
4. CPCM-RPUF composites with higher FS content (23% and 25%) prevent PCM leakage and maintain or improve compressive strength compared to pure PUs.

## Figures and Tables

**Table 1:** TG data of pure PUs and CPCM-RPUF in nitrogen gas.

Sample	T_5%_ (°C)	T_10%_ (°C)	T_50%_ (°C)	T_DTGmax1_	T_DTGmax2_	T_DTGmax3_	Residue at 600 °C (%)
pure PU	270	301	396	334	401	500	23
CPCM-RPUF-1	254	292	404	319	396	484	34
CPCM-RPUF-2	291	327	411	-	381	493	34
CPCM-RPUF-3	302	328	430	357	411	493	40
CPCM-RPUF-4	303	329	518	346	403	480	46
CPCM-RPUF-5	285	309	561	303	402	562	47

## Data Availability

The original contributions presented in this study are included in the article. Further inquiries can be directed to the corresponding author.
